# Supplementary material for: Heat adaptation of phage T7 under an extended genetic code
Source: Virus Evol. 2021 Dec 1;7(2):veab100. doi: 10.1093/ve/veab100 (PMC8923235; doi:10.1093/ve/veab100)
Supplement: veab100_Supp [file veab100_supp.zip › Sequencing_Analysis.zip!/Sequencing_Analysis/A1/evidence/JC_SIDE_2_57.html]

BRESEQ :: Evidence


| New junction evidence | | | | | | | | | | | |
| --- | --- | --- | --- | --- | --- | --- | --- | --- | --- | --- | --- |
|  | | seq id | position | reads (cov) | reads (cov) | score | skew | freq | annotation | gene | product |
| \* | ? | NC\_001604.1 | = 13277 | 2147 (0.700) | 671 (0.220) | 245/586 | 2.8 | 38.4% | intergenic (–/–) | *–/–* | –/– |
|  | ? | NC\_001604.1 | 14297 = | 53 (0.020) |  |  |  |  | intergenic (–/–) | *–/–* | –/– |

Only 100 of 733 total aligned reads displayed.  

`ATGAAGCTAAGACTCGCTCCGCTGTTCACATCTTGAAGAACTTAGGCTGGACTTGGACAAGACAGACTGGCTGGAAGAAACCAGAAGTTACCAAGCTGAGTCATAAGGTGTTCGATAAGGACACTATGACCCACATCAAGGCTGGTGATTGGGTTAAGGTTGACATGGGAGTTGTTGGTGGATACGGCTACGTCCGCTCAGTTAGTGGCAAATATGCACAAGTGTCATACATCACAGGTGTTACTCCACGCGGTGCAATCGTTGCCGATAAGACCAACATGATTCACACAGGTTTCTTGACAGTTGTTTCATATGAAGAGATTGTTAAGTCACGATAATC‑AATAGGAGAAATCAATATGATCGTTTCTGACATCGAAGCTAACGCCCTCTTAGAGAGCGTCACTAAGTTCCACTGCGGGGTTATCTACGACTACTCCACCGCTGAGTACGTAAGCTACCGTCCGAGTGACTTCGGTGCGTATCTGGATGCGCTGGAAGCCGAGGTTGCACGAGGCGGTCTTATTGTGTTCCACAACGGTCACAAGTATGACGTTCCTGCATTGACCAAACTGGCAAAGTTGCAATTGAACCGAGAGTTCC  >  NC_001604.1/13997‑14596`  

                                                                                                                                                                                                                                                                                                            `|`                                                                                                                                                                                                                                                                                                              
`gtgtataagagacaggaaggtcgccccgtttctattactgacctacgtggttctggcgcactacgccaactatctgatactattattgcccttgagcgtaatcagcaaggcgatatgcctaaccttgtcctcgttcgtattctcaagtgccgctttactggtgatactggtatcgctggctacatggaatacaacaaggaaaccggatggcttgaaccatcaagttactcaggggaagaagagtcacactcagagtcaacagactggtccaacgacactgacttctgacaggattcttgac                                                                                                                                                                                                                                                                                                              <  1:408935‑M2/1‑1 (MQ=255)`  
`ggtaaagcacatgaggaaggtcgccccgtttctattactgacctacgtggttctggcgcactacgccaactatctgatactattattgcgcttgagcgtaatcagcaaggcgatatgcctaaccttgtcctcgttcgtattctcaagtgccgctttactggtgatactggtatcgctggctacatggaatacaacaaggaaaccggatggcttgaaccatcaagttactcagtggaataagagtcacactcagagtcaacagactggtcccacgacactgacttcttacaggaatattgac                                                                                                                                                                                                                                                                                                              >  1:42427‑M2/301‑301 (MQ=255)`  
  `aaaagcaccttatgaaggtcgccccgtttctattactgacctacgtggttctggcgcactacgccaactagctgatactattattgcccgtgagcgtaatcagcaaggcgatatgcctaaccttgtcctcgttcgtattcgcaagggccgctttacgggtgatactggtatcgctggcgacatggaatacaacaaggaaaccggatggcttgaaccatcaagttactcaggggaagaagagtcacactcagagtcaacagactggtccaacgacactgacttctgacaggattcttgaCAg                                                                                                                                                                                                                                                                                                            <  1:192630‑M2/3‑1 (MQ=255)`  
       `cacatgaggaaggtcgccccgtttctattactgacctacgtggttctggcgcactacgccaactatcttatactattattgcccttgagcgtaatcagcaaggcgatatgcctaaccttgtcctcgtacgtattctcaagtgccgctttactggtgatactggtatcgctggctacatggaatacaacaaggaaaccggatggcttgaaccatcaagttactcaggggaagaagagtcacactcagagtcaacagactggtccaacgacaccgacttctgacaggattcttgaCAgttgtt                                                                                                                                                                                                                                                                                                       >  1:110205‑M2/294‑301 (MQ=255)`  
                 `aggtcgccccgtttctattactgacctacgtggttctgtcgcactacgccaactatctgatacgattattgcccttgagcgtaatcagcaaggcgatatgcctaaccttgtcctcgttcgtattctcaagtgccgctttactggtgatactggtatcgctggctacatggaatacaacagggaaaccggatggcttgaaccatcaagttactcaggggaagaagagtcacactcagagtcaacagactggtccaacgacactgacttctgacaggattcttgaCAGTTGTTTCATATGAag                                                                                                                                                                                                                                                                                             <  1:122926‑M2/18‑1 (MQ=255)`  
                 `aggtcgccccggttctattactgacctacgtggttctggcgcactacgccaactatctgatactattattgcccttgagcgtaatcagcaaggcgatatgcctaaccttgtcctcgttcgtattctcaagtgccgctttactggtgatactggtatcgctggcgacatggaaaacaacaaggaaaccggatggcttgaaccatcaagttactcaggggaagaagagtcacactcagagtcaacagactggtccaacgacactgacttctgacaggattcttgaCAGTTGTTTCATATGAag                                                                                                                                                                                                                                                                                             <  1:235423‑M2/18‑1 (MQ=255)`  
                      `tGTTCACATCTTGAAGAACTTAGGCTGGACTTGGACAAGACAGACTGGCTGGAAGAAACCAGAAGTTACCAAGCTTAGTCATAAGGTGTTCGATAAGGACACTATGACCCACATCAAGGCTGGTGATTGGGTTAAGGTTGACATGGGAGTTGTTGGTGGATACGGCTACGTCCGCTCAGTTAGTGGCAAATATGCACAAGTGTCATACATCACAGGTGTTACTCCACGCGGTGCAATCGTTGCCGATAAGACCAACATGATTCACACAGGTTTCTTGACAGTTGTTTCATATGAAGAGAtt                                                                                                                                                                                                                                                                                        <  1:88410/301‑1 (MQ=255)`  
                            `tttctattactgacctacgtggttctgtcgcactacgccaactatctgatactattatttccctttagcgtaatcagcaagtcgctatgcctaaccttgttctcgttcgtattctcaagtgccgctttactggttattcttgtatcgctggctacatgctattccacaaggaaaccggatggcttgaaccatcaagttactcaggggaagaagagctccactcagtgtcaacagacttgtccaacgacactgacttctgacaggattcttgaCAGTTGTATCATTTGAATAGATTTttatg                                                                                                                                                                                                                                                                                  >  1:470716‑M2/273‑299 (MQ=255)`  
                               `ttattactgacctacgtggttctggcgcactacgccaactatctgatactattattgcccttgagcgtaatcagcaaggcgatatgcctaaccttgtcctcgttcgtattctcaagtgccgctttactggtgatactggtatcgctggctacatggaatacaacaaggaaaccggatggcttgaaccatcaagttactcaggggaagaagagtcacactcagagtcaacagactggtccaacgacactgacttctgacaggattcttgaCAGTTGTTTCATATGAAGAGATTGTTAAGTCa                                                                                                                                                                                                                                                                               <  1:4164‑M2/32‑1 (MQ=255)`  
                                   `tggatgaagagtcacactcagagtcaacagacTGGCTGGAAGAAACCATAAGTTACCAAGCTTAGTCATAAGGTGTTCGATAAGGACACTATGACCCACATCAAGGCTGGTGATTGGGTTAAGGTTGACATGGGAGTTGTTGGTGGATACTGCTACGTCCGCTCAGTTAGTGGCAAATATGCACAAGTGTCATACATCACAGGTGTTACTCCACGCGGTGCAATCGTTGCCGATAAGACCAACATGATTCACACAGGTTTCTTGACAGTTGTTTCATATGAAGAGATTGTTAAGTCACGAt                                                                                                                                                                                                                                                                           <  1:148349/275‑1 (MQ=255)`  
                                     `gaagaagagtcacactcagagtcaacagacTGGCTGGAAGAAACCAGAAGTTACCAAGCTGAGTCATAAGGTGTTCGATAAGGACACTATGACCCACATCAAGGCTGGTGATTGGGTTAAGGTTGACATGGGAGTTGTTGGTGGATACGGCTACGTCCGCTCAGTTAGTGGCAAATATGCACAAGTGTCATACATCACAGGTGTTACTCCACGCGGTGCAATCGTTGCCGATAAGACAAACATGATTCACACAGGTTTCTTGACAGTTGTTTCATATGACAAGATTGTTAAGTCACGATaa                                                                                                                                                                                                                                                                         >  1:293159/25‑301 (MQ=255)`  
                                        `tataagagacagctggcgcactacgccaactatctgatactattattgcccttgagcgtaatcagcaaggcgatatgcctaaccttgtcctcgttcgtattctcaagtgccgcgttactggtgatactggtatcgctggctacatggaatacaacaaggaaaccggatggcttgaaccatcaagttactcaggggaagaagagtcacactcagagtcaacagactggtccaacgacactgacttctgacaggattcttgaCAGTTGTTTCATATGAAGAGATTGTTAAGTCACGATAATC‑a                                                                                                                                                                                                                                                                     <  1:329002‑M2/41‑1 (MQ=255)`  
                                            `acgtggttctggcgcactacgccaactatctgatactattattgcccttgagcgtaatcagcaaggcgatatgcctaaccttgtcctcgttcgtattctcaagtgccgctttactggtgatactggtatcgctggctacatggaatacaacaaggaaaccggatggcttgaaccatcaagttactcatgggaagaagagtcacactcagagtcaacagactggtccaacgacactgacttctgacagttttcttgaCAGTTGTTTCATCTGACGAGATTGTTAAGTCACGATAATC‑AATAg                                                                                                                                                                                                                                                                 >  1:66731‑M2/257‑301 (MQ=255)`  
                                             `cgtggttccgccgcaatacgccaactatctgataatatgattgcccttgagcgtaatcagcaaggcgatatgcctaaccttgtcctcgttagtattctcacgtgccgctttactggtgatactggtatcgctggctacatggaatacaacaaggaaaccggatggcttgatccataacgttactcaggggaagagaagtcacaatcagagtcaacagacttgtccagcgacactgacttctgacaggattcttgaCAGTGATTTCATATGAAGAGATTGTTAAGTCACGATAATC‑AATAgg                                                                                                                                                                                                                                                                <  1:94506‑M2/46‑1 (MQ=255)`  
                                               `cacactaagagtcaacggacTGGATGGAAGAAACCAAAAGTTACCAAGTTGATTCATAAGGTGTTCGATAAGGACACTATGACCAACATCAAGGCTGGTGATTGGGTTAAGGTTGACATGGGAGTTGTTGTTGGATACGGCTACTCCTGCTCATTTAGTGGCAAATATGCACAAGTGTCATACATCACAGGTGTTACTCCACGCGGTGCAATCGTTGCCGATAAGACCAACATGATTCACACAGGTTTCTTGACAGTTGTTTCATATGAAGAGATTGTTAAGTCACGATAATC‑AATAGgag                                                                                                                                                                                                                                                              <  1:302816/284‑1 (MQ=255)`  
                                                 `gttctggcgcactacgccaactatctgatactattattgcccttgagcgtaatcagcaaggcgatatgcctaaccttgtcctcgttcgtattctcaagtgccgctttactggtgatactggtatcgctggctacatggaatacaacaaggaaaccggatggcttgaaccatcaagttactcaggggaagaagagtcacactcagagtcaacagactggtccaacgacactgacttctgacaggattcttgaCAGTTGTTTCATATGAAGAGATTGTTAAGTCACGATAATC‑AATAGGAGaa                                                                                                                                                                                                                                                            >  1:251744‑M2/252‑301 (MQ=255)`  
                                                            `ctacgccaactatctgatactattattgcccttgagcgtaatcagcaaggcgatatgcctaaccttgtcctcgttcgtattctcaagtgccgctttactggtgatactggtatcgctggctacatggaatacaacaaggaaaccggatggcttgaaccatcaagttactcaggggaagaagagtcacactcagagtcaacagactggtccaacgacactgacttctgacaggattcttgaCAGTTGTTTCATATGAAGAGATTGTTAAGtcacgataatc‑aataggctgtctcttatacac                                                                                                                                                                                                                                                 >  1:396847‑M2/241‑286 (MQ=255)`  
                                                            `atacgccaacgatgtgatactattattgcccttgagcgtaatcagaaaggcgatatgcctaaccttgtcctcgttcgtattctcaagtgccgctttactggtgatactggtatcgctggctacatggaatacaacaaggaaaccggatggcttgaaccatcaagttactcaggggaagaagagtcacactcagagtcaacagactggtccaacgaaactgacttctgacaggtttcttgaCAGTTGTTTCATATGAAGAGATTGTTAAGTCACGATAATC‑AATAGGAGAAATCAATATGAt                                                                                                                                                                                                                                                 <  1:431362‑M2/61‑1 (MQ=255)`  
                                                                 `ccaactatctgatactattattgcccttgagcgtaatcagcaaggcgatatgcctaaccttgtcctcgttcgtattctcaagtgccgctttactggtgatactggtatcgctggctacatggaatacaacaaggaaaccggatggcttgaaccatcaagttactcaggggaagaagagtcacactcagagtcaacagactggtccaacgacactgacttctgacaggattcttgaCAGTTGTTTCATATGAAGAGATTGTTAAGTCACGATAATC‑AATAGGAGACATCAATATGATCGttt                                                                                                                                                                                                                                            >  1:406815‑M2/236‑301 (MQ=255)`  
                                                                  `atgctatcttatactcttattgacctttagcgtaatcagaaaggcgatatgcctaaccttgtcctcgttcgtagtctcaagtgccgctttactggtgatactggtatcgctggctacatggaatacaacaaggaaaccggatagcttgaaccatcaagttactcaggggaagaagagtcacactaagagtcaacagactggtccaacgacactgacttctgacaggattcttgaCAGTTGTTTCATATGAAGAGATTGTTAAGTCACGATAATC‑AATAGGAGAAATCAATATGATCGTTTc                                                                                                                                                                                                                                           <  1:48523‑M2/67‑1 (MQ=255)`  
                                                                       `taagagacagattattgcccttgagcgtaatcagcaaggcgatatgcctaaccttgtcctcgttcgtattctcaagtgccgctttactggtgatactggtatcgctggctacatggaatacaacaaggaaaccggatggcttgaaccatcaagttactcaggggaagaagagtcacactcagagtcaacagactggtccaacgacactgacttctgacaggattcttgaCAGTTGTTTCATATGAAGAGATTGTTAAGTCACGATAATCAAACAGGAGAAATCAATATGATCGTTTCTGAc                                                                                                                                                                                                                                       <  1:477915‑M2/72‑1 (MQ=255)`  
                                                                       `atctgatactattattgcccttgagcgtaatcagcaaggcgatatgcctaaccttgtcctcgttcgtattctcaagtgccgctttactggtgatactggtatcgctggctacatggaatacaacaaggaaaccggatggcttgaaccatcaagttactcaggggaagaagagtcacactcagagtcaacagactggtccaacgacactgacttctgacaggattcttgaCAGTTGTTTCATATGACGAGATTGTTAAGTCACGATAATC‑AATAGGAGAAATCAATATGATCGTTTCTGACa                                                                                                                                                                                                                                      >  1:444549‑M2/230‑301 (MQ=255)`  
                                                                       `atctgatactattattgcccttgagcgtaatcagcaaggcgatatgcctaaccttgtcctcgttcgtattctcaagtgccgctttactggtgatactggtatcgctggctacatggaatacaacaaggaaaccggatggcttgaaccatcaagttactcaggggaagaagagtcacactcagagtcaacagactggtccaacgacactgacttctgacaggattcttgaCAGTTGTTTCATATGAAGAGATTGTTAAGTCACGATAATC‑AATAGGAGAAATCAATATGATCGTTTCTGACa                                                                                                                                                                                                                                      >  1:412510‑M2/230‑301 (MQ=255)`  
                                                                                 `attattgcccttgagcgtaatcagcaaggcgatatgcctaaccttgtcctcgttcgtattctcaagtgccgctttactggtgatactggtatcgctggctacatggaatacaacaaggaaaccggatggcttgaaccatcaagttactcaggggaagaagagtcacactcagagtcaacagactggtccaacgacactgacttctgacaggattcttgaCAGTTGTTTCATATGAAGAGATTGTTAAGTCACGATAATC‑AATAGGAGAAATCAATATGATCGTTTCTGACATCGAAGCTaa                                                                                                                                                                                                                            >  1:107422‑M2/220‑301 (MQ=255)`  
                                                                                 `attattgcccttgagcgtaatcagcaaggcgatatgcctaaccttgtcctcgttcgtattctcaagtgccgctttactggtgatactggtatcgctggctacatggaatacaacaaggaaaccggatggcttgaaccatcaagttactcaggggaagaagagtcacactcagagtcaacagactggtccaacgacactgacttctgacaggattcttgaCAGTTGTTTCATATGAAGAGATTGTTAAGACACGATAAGC‑AATAGGAGAAATCATAATGATCGTTTTTGACATCGAAGCTaa                                                                                                                                                                                                                            >  1:492913‑M2/220‑301 (MQ=255)`  
                                                                                 `attattgcccttgagcgtaatcagcaaggcgatatgcctaaccttgtcctcgttcgtattctcaagtgccgctttactggtgatactggtatcgctggctacatggaatacaacaaggaaaccggatggcttgaaccatacagttactcaggggaagaagagtcacactcagagtccacacactggtccaacgacactgacttctgacaggattcttgaCAGTTGTTTCATATGAAGAGAGTGTTAAGTCACGATAATA‑AATAGGAGAAATCAATATGATCGTTTCTGACATCGACGCTaa                                                                                                                                                                                                                            >  1:174936‑M2/220‑301 (MQ=255)`  
                                                                                          `cttgagcgtaatcagcaaggcgatatgcctaaccttgtcctcgttcgtattctcaagtgccgctttactggtgatactggtatcgctggctacatggaatacaacaaggaaaccggatggcttgaaccatcaagttactcaggggaagaagagtcacactcagaggcaacagactggtctaacgacaatgcattctgacaggattcttgaCAGTTGTTCTATATGAAGAGATTGTTAAGTCACGATAATC‑AATAGGAGAAATAAATATGATCGTTTCTGACATAGAAGCTAACgcgctggc                                                                                                                                                                                                                   >  1:136542‑M2/211‑298 (MQ=255)`  
                                                                                              `ttcgtaatcagcaaggcgatatgcctaaccttgtcctcgttcgtattctcaagtgccgctttactggtgatactggtttcgctggctacatggaatacaacaaggaaactggatggcttgaaccatcaagttactcaggggaagaagagtcacactcagagtcaacagactggtccaacgacactgacttctgacaggattcttgaCAGTTGTTTCATATGAAGAGATTGTTAAGTCACGATAATC‑AATAGGAGAAATCAATATGATCGTTTCTGACATCGAAGCTAACGCCCTCTTagag                                                                                                                                                                                                               <  1:437262‑M2/95‑1 (MQ=255)`  
                                                                                              `ggcgtaatcagcaaggcgatatgcctaaccttgtcctcgttcgtattctcaagtgccgctttactggtgatactggtatcgctggctacatggaatacaacaaggaaaccggatggcttgaaccatcaagttactcaggggaagaagagtcacactcagagtcaacagactggtccaacgacactgacttctgacaggattcttgaCAGTTGTTTCATATGAAGAGATTGTTAAGTCACGATAATC‑AATAGGAGAAATCAATATGATCGTTTCTGACATCGAAGCTAACGCCCTCTTagag                                                                                                                                                                                                               <  1:8548‑M2/95‑1 (MQ=255)`  
                                                                                              `agcgtaatcagcaagtcgatatgcctaaccttgtcctcgttcgtattcgcaagtgccgctttactggtgatactggtatcgctggctacatggaatacaacaaggaaaccggatggcttgaaccatcaagttactcaggggaagaagagtcacactcagagtcaacagactggtccaacgacactgacttctgacaggattcttgaCAGTTGTTTCATATGAAGAGATTGTTAAGTCACGATAATC‑AATAGGAGAAACCATTATGATCGTTTCTGACATCGAAGCTAACGCCCTCTTagag                                                                                                                                                                                                               <  1:296709‑M2/95‑1 (MQ=255)`  
                                                                                                `ggtaataagcaaggcgatatgcctaacattgtcctcgttcgtattctcaagtgccgctttactggtgatactggtatcgctggctacatggaatacaacaaggaaaccggatggcttgaaccatcaagttactcaggggaagaagagtcacactcagagtcaacagactggtccaacgacactgacttctgacaggattcttgaCAGTTGTTTCATATGAAGAGATTGTTAAGTCACGATAATC‑AATAGGAGAAATCAATATGATCGTTTCTGACATCGAAGCTAACGCCCTCTTagagag                                                                                                                                                                                                             <  1:216748‑M2/97‑1 (MQ=255)`  
                                                                                                `gagatgtgtataagagacaggcctaaccgtgtcctcgttagtattctcaagtgccgctttactggtgatactggtatcgctggctacatggaatacaacaaggaaaccggatggcttgaaccatcaaggtactcaggggaagaagagtcacactcagagtcaacagactggtccaacgacactgacttctgacaggattcttgaCAGTTGTTTCATATGAAGAGATTGTTAAGTCACGATAATC‑AATAGGAGAAATCAATATGATCGTTTCTGACATCGAAGCTAACGCCCTCTTagagag                                                                                                                                                                                                             <  1:400962‑M2/97‑1 (MQ=255)`  
                                                                                                 `gtaatcagcaaggcgatatgcctaaccttgtcctcgttcgtattctcaagtgccgctttactggtgatactggtatcgctggctacatggaatacaacaaggaaaccggatggcttgaaccatcaagttactcagggaaagaagagtcacactcagagtcaacagactggtccaacgacactgacttctgacaggattcttgaCAGTTGTTTCATATGAAGAGATTGTTAAGTCACGATAATC‑AATAGGAGAAATCAATATGATCGTTTCTGACATCGAAGCTAACGCCCTcttagagctg                                                                                                                                                                                                            >  1:231204‑M2/204‑298 (MQ=255)`  
                                                                                                  `taatcagcaaggcgatatgcctaaccttgtcctcgttcgtattctcaagtgccgctttactggtgatactggtatcgctggatacatggaatacaacaaggaaaccggatggcttgaaccatcaagttactcaggggaagaagagtcacactcagagtcaacagactggtccaacgacactgacttctgacaggattcttgaCAGTTGTTTCATATGAAGAGATTGTTAAGTCACGATAATC‑AATAGGAGAAATCAATATGATCGTTTCTGACATCGAAGCTAACGCCCtcttagagctgt                                                                                                                                                                                                           >  1:72772‑M2/203‑297 (MQ=255)`  
                                                                                                    `atcagcaaggcgatatgcctaaccttgtcctcgttcgtattctcaagtgccgctttactggtgatactggtatcgctggctacatggaatacaacaaggaaaccggatggcttgaaccatcaagttactcaggggaagaagagtaacactcagagtcaacagactggtccaacgacactgacttctgacaggattcttgaAAGTTGTTTCATATGAAGAGATTGTTAAGTCACGATAATC‑AATAGGAGAAATCAATATGATCGTTTCTGACATCGAAGCTAACGCactcttagagcggtct                                                                                                                                                                                                         >  1:82388‑M2/201‑295 (MQ=255)`  
                                                                                                          `ggTGTTCGATAAGGACACTATGACCCACATCAAGGCTGGTGATTGGGTTAAGGTTGACATGGGAGTTGTTGGTGGATACGGCTACGTCCGCTAAGTTAGTGGCAAATATGCACAAGTGTCATACATCACAGGTGTTACTCCACGCGGTGCAATCGTTGCCGATAAGACCAACATGATTCACACAGGTTTCTTGACAGTTGTTTCATATGAAGAGATTGTTAAGTCACGATAATC‑AATAGGAGAAATCAATATGATAGTTTCTGACATCGAAGCTAACGCCCTCTTAGAGAGCGTAACTact                                                                                                                                                                                                   >  1:36146/1‑299 (MQ=255)`  
                                                                                                          `aaggctataagcctaaccttgtcctctttcgaatgctaaagtgccgatgtaatggggataatggtatcgctggctacatggaatacaacaaggaaaccggatggcttgaaccatcaagttactcaggggaagaagagtcacactcagagtcaacagactggtccaacgaaactgactgctgacaggattcttgaTAGTGGTTTCATAGGAAGAGATTGTTAAGTCACGATAATC‑AATAGGAGAAATCAATATGATCGTTTCTGACATCGAAGCTAACGCCCTCTTAGAGAGCGTCACTAAg                                                                                                                                                                                                   <  1:247266‑M2/107‑1 (MQ=255)`  
                                                                                                              `ggatatgcctaaccttgtcctagttagtattcgcaagtgccgctttactggtgatactggtatcgcgggctacatggaatacaacaaggaaaccggatggcttgaaccatcaagttactcaggggaagaagagtcacactcagagtcaacagactggtacaacgacactgacttctgacaggattcttgaCAGTTGTTTCATATGAAGAGAGTGTTAAGTCACGATAATC‑AATAGGAGAAATCAATATGATCGTTTCTGACATCGAAGCTAACGCCCTCTTAGAGAGCGTCACTAAGTTcc                                                                                                                                                                                               <  1:162367‑M2/111‑1 (MQ=255)`  
                                                                                                              `cgatatgccttaccttgtcctcgttcgtattctcaagtgactctttactggtgatactggtatcgctggctacatggaatacaacaaggaaaccggatggcttgaaccatcaagttactcaggggaagaagagtcacactcagagtcaacagactggtccaacgacactgacttctgacaggattcttgaCAGTTGTTTCATATGAAGAGATTGTTAAGTCACGATAATC‑AATAGGAGAAATCAATATGATAGTTTCTGACATCGAAGCTAACGCCCTCTTAGAGAGCGTCACTAAGTTcc                                                                                                                                                                                               <  1:271956‑M2/111‑1 (MQ=255)`  
                                                                                                               `gatatgcctaaccttgtcctcgttcgtattctcaagtgccgctttactggtgatactggtatcgctggctacatggaatacaacaaggaaaccggatggcatgaaccatcaagttactaaggggaagaagagtcacactcagagtcaacagactggtccaacgacactgacttctgacaggattcttgaCAGTTGTTTCATATGAAGAGATTGTTAAGTCACGATAATC‑AATAGGAGAAATCAATATGATCGTTTCTGACATCGAAGCTACCGCCCTCTTAGAGAGCGTCACTAAGTTCCa                                                                                                                                                                                              >  1:266423‑M2/190‑301 (MQ=255)`  
                                                                                                                    `gcctaaccttgtcctcgttcgtattctcaagtgccgctttactggtgatactggtatcgctggctacatggaatacaacaaggaaaccggatggcttgaaccatcaagttactcaggggaagaagagtcacactcagagtcaacagactggtccaacgacactgacttctgacaggattcttgaCAGTTGTTTCATATGAAGAGATTGTTAAGTCACGATAATC‑AATAGGAGAAATCAATATGATCGTTTCTGACATCGAAGCTAACGCCCTCTTAGAGAGCGTCACTAAGTTCCACTGCg                                                                                                                                                                                         >  1:374589‑M2/185‑301 (MQ=255)`  
                                                                                                                    `gcctaaccttgtcctcgttcgtattctcaagtgcagctttactggtgatactggtatcgctggctacatggaatacaacaaggaaaccggatggcttgaaccatcaagttactcaggggaagaagagtcacactcagagtcaacagactggtccaacgacactgacttctgacaggattcttcaCAGTTGTTTCATATGAAGAGATTGTTAAGTCACGATAATC‑AATAGGATAAATCAATATGATCGTTTCTGACATCGAATCTAACGCCctcttagagacggtcatcaattaccacctgt                                                                                                                                                                                         >  1:78473‑M2/185‑286 (MQ=255)`  
                                                                                                                        `aaccgtgtcctcgttcgtattctcaagtgccgctttactggtgatactggtatcgctggctacatggaatacaacaaggaaaccggatggcttgaaccatcaagttactcaggggaagaagagtcacaatcagagtcaacagactggtccaacgacactgacttctgacaggattcttgaCAGTTGTTTCATATGAAGAGATTGTTAAGTCACGATAATC‑AATAGGAGAAATCAATATGATCGTTTCTGACATCGAAGCTAACGCCCTCTTAGAGAGCGTCACTAAGTTCCACTGCGGGGt                                                                                                                                                                                     <  1:151708‑M2/121‑1 (MQ=255)`  
                                                                                                                         `accttgtcctcgttcgtattatcaagtgccgctttactggtgatactggtatcgctggctacatggaatacaacaaggaaaccggatggcttgaaccatcaagttactcaggggaagaagagtcacactcagagtcaacagactggtccaacgacactgacttctgacaggattcttgaCAGTTGTTTCATATGAAGAGATTGTTAAGTCACGATAATA‑AATAGGAGAAATCAATATGATCGTTTCTGACATCGAAGCTAACGCCCTCTTAGAGAGCGTCACTAAGTTCCACTGCGGGGtt                                                                                                                                                                                    >  1:179839‑M2/180‑301 (MQ=255)`  
                                                                                                                                  `tcgttcgtattctcaagtgccgctttactggtgatactggtatcgctggctacatggaatacaacaaggaaaccggatggcttgaaccatcaagttactcaggggaagaagagtcacactcagagtcaacagactggtccaacgacactgacttctgacaggagtcttgaCAGTTGTTTCATATGAAGAGATTGTTAAGTCACGATAATC‑AATAGGAGAAATCAATATGATCGTTTCTGACATCGAAGCTAACGCCCTCTTAGAGAGCGTCACTAAGTTCCACTGCGGGGTTATCTACGac                                                                                                                                                                           <  1:241407‑M2/131‑1 (MQ=255)`  
                                                                                                                                        `ttgtataagtgccaggctttactggtgatactggtatcgcgggctacatggaatacaacaaggaaaccggatggcgtgaaccatcaagttactcaggggaagaagagtcacactcagagtaaacagactggtccaacgacactgacatctgacaggattcttgaCAGTTGTTTCATATGAAGAGATTGTTAAGTCACGATAATC‑AATAGGAGAAATCAATATGATAGTTTCTGACATCGAAGCTAACGCCCTCTTAGAGAGCGTCACTAAGTTCCACTGCGGGGTTATCTACGACTACTcc                                                                                                                                                                     <  1:16756‑M2/137‑1 (MQ=255)`  
                                                                                                                                             `ctcaagtgccgctttactggtgatactggtatcgctggctacatggaatacaacaaggaaaccggatggcttgaaccatcaagttactcaggggaagaagagtcacactcagagtcaacagactggtccaacgacactgacttctgacaggattcttgaCAGTTGTTTCATATGAAGAGAGTGTTAAGTCACGATAATC‑AATAGGAGAAATCAATATGATCGTTTCTGACATCGAAGCTAACGCCCTCTTAGAGAGCGTCACTAAGTTCCACTGCGGGGTTATCTACGACTACTCCACCGc                                                                                                                                                                <  1:463000‑M2/142‑1 (MQ=255)`  
                                                                                                                                               `cgttcgtattctcaagtgccgctttactggtgatactggtatcgctggctacatggaatacaacaaggaaaccggatggcttgaaccatcaagttactcaggggaagaagagtcacactcagagtcaacagactggtccaacgacactgacttctgaCAGTTGTTTCATATGAAGAGATTGTTAAGTCACGATAATC‑AATAGGAGAAATCAATATGATCGTTTCTGACATCGAAGCTAACGCCCTCTTAGAGAGCGTCACTAAGTTCCACTGCGGGGTTATCTACGACTACTCCACCGCTg                                                                                                                                                              <  1:189819‑M2/144‑1 (MQ=255)`  
                                                                                                                                                             `aaagtgatactggtatctctggctacatggaatacaacaaggaaaccggatggcttgaaccatcaagttactcaggggaagaagagtaacactcagagtcaacagactggtccaactacactgacttctgacaggattcttgaCAGTTGTTTCATATGAAGAGATTGTTAAGTCACGATAATC‑AATAGGAGAAATCAATATGATCGTTTCTGACATCGAAGCTAACGCCCTCTTAGAGAGCGTCACTAAGTTCCACTGCGGGGTTATCTACGACTACTCCACCGCTGAGTACGTAAGCTAc                                                                                                                                                <  1:158028‑M2/158‑1 (MQ=255)`  
                                                                                                                                                              `tggtgatactggtatcgctggctacatggaatacaacaaggaaaccggatggcttgaaccatcaagttacgcaggggaagaagagtcacactcagagtcaacagactggtccaacgacactgacttctgacaggattcttgaCAGTTGTTTCATATGAAGAGATTGTTAAGTCACGATAATC‑AATAGGAGAAATCAATATGATCGTTTCTGACATCGAAGCTAACGCCCTCTTAGAGAGCGTCACTAAGTTCCACTGAGGGGTTATCTACGACTACTCCACCGCTGTGTACGTAAGCTAcc                                                                                                                                               >  1:60413‑M2/143‑301 (MQ=255)`  
                                                                                                                                                               `aacgtacagggtatcgcgggctaaatggactacaacaaggaaaccggatggcttgaaccatcaagttactcaggggaagaagagtcacactcagagtcaacagactggtccaacgacacttacttctgacaggtttcttgaCAGTTGTTTCATATGAAGAGATTGTTAAGTCACGATAATC‑AATAGGAGAAATCAATATGATCGTTTCTGACATCGAAGCTAACGCCCTCTTAGAGAGCGTCACTAAGTTCCACTGCGGGGTTATCTACGACTACTCCACCGCTGAGTACGTAAGCTACCg                                                                                                                                              <  1:337958‑M2/160‑1 (MQ=255)`  
                                                                                                                                                                `gtgatactggtatcgctggctacatgtaatacaacaaggaaaccggatggcttgaaccatcaagttactcaggggaagaagagtcacactcagagtcaacagactggtccaacgacactgacttctgacaggattcttgaCAGTTGTTTCATATGAAGAGATTGTTAAGTCACGATAATC‑AATAGGAGAAATCAATATGATCGTTTCTGACATCGAAGCTAACGCCCTCTTAGAGAGCGtcactaagttccactgcggggttatctacgacctgtctcttatacacatctcccgagcccac                                                                                                                                             >  1:130953‑M2/141‑271 (MQ=255)`  
                                                                                                                                                                        `ggtatcgctggctacatggaatacaacaaggaaaccggatggcttgaaccatcaagttactcaggggaagaagagtcacactcagagtcaacagactggtccaacgacactgacttctgacaggattcttgaCAGTTGTTTCATATGAAGAGATTGTTAAGTCACGATAATC‑AATAGCAGAAATCAATATGATCGTTTCTGACATCGAAGCTAACGCCCTCTTAGAGAGCGTCACTAAGTTCCACTGCGGGGTTATCTACGACTACTCCACCGCTGAGTACGTAAGCTACCGTCCGAGTGa                                                                                                                                     >  1:325382‑M2/133‑301 (MQ=255)`  
                                                                                                                                                                            `ggctcggagatgtgtataagagacaggaatacaacaaggaaaccggatggcttgaaccatcaagttactcaggggaagaagagtcacactcagagtcaacagactggtccaacgacactgacttctgaCAGTTGTTTCATATGAAGAGATTGTTAAGTCACGATAATC‑AATAGGAGAAATCAATATGATCGTTTCTGACATCGAAGCTAACGCCCTCTTAGAGAGCGTCACTAAGTTCCACTGCGGGGTTATCTACGACTACTCCACCGCTGAGTACGTAAGCTACCGTCCGAGTGACTTc                                                                                                                                 <  1:475036‑M2/173‑1 (MQ=255)`  
                                                                                                                                                                             `cgctggctacatggaatacaacaaggaaaccggatggcttgaaccatcaagttactcaggggaagaagagtcacactcagagtcaacagactggtccaacgacactgacttctgacaggattcttgaCAGTTGTTTCATATGAAGAGATTGTTAAGTCACGATAATC‑AATAGGAGAAATCAATATGATCGTTTCTGACATCGAAGCTAACGCCCTCTTAGAGAGCGTCACTAAGTTCCACTGCGGGgttatctacgactactccaccgctgagtccctgtctcttatccacatctccgagc                                                                                                                                >  1:319562‑M2/128‑274 (MQ=255)`  
                                                                                                                                                                              `tataagagacaggaatacaacaaggaaaccggatggcttgaaccatcaagttactcaggggaagaagagtcacactcagagtcaacagactggtccaacgacactgacttctgacaggattcttgaCAGTTGTTTCATATGAAGAGATTGTTAAGTCACGATAATC‑AATAGGAGAAATCAATATGATCGTTTCTGACATCGAAGCTAACGCCCTCTTAGAGAGCGTCACTAAGTTCCACTTCGGGGTTATCTACGACTACTCCACCGCTGAGTACGTAAGCTACCGTCCGAGTGACTTCgg                                                                                                                               <  1:144866‑M2/175‑1 (MQ=255)`  
                                                                                                                                                                                `gtgtataagagaaagcaacaaggaaaccggatggcttgaaccatcaagttaatcaggggaagaagagtcacactcagagtcaacagactggtccaacgacactgacttctgacaggattcttgaCAGTTGTTTCATATGAAGAGATTGTTAAGTCACGATAATC‑AATAGGAGAAATCAATATGATCGTTTCTGACATCGAAGCTAACGCCCTCTTAGAGAGCGTAACTAAGTTCCACTGCGGGGTTATCTACGACTACTCCACCGCTGAGTACGTAAGCTACCGTCCGAGTGACTTCGGTg                                                                                                                             <  1:185801‑M2/177‑1 (MQ=255)`  
                                                                                                                                                                                  `gctacatggaatacaacaaggaaaccggatggcttgaaccatcaagttactcaggggaagaagagtcacactcagagtcaacagactggtccaacgacactgacttctgacaggattcttgaCAGTTGTTTCATATGAAGAGATTGTTAAGTCACGATAATC‑AATAGGAGAAATCAATATGATCGTTTCTGACATCGAAGCTAACGCCCTCTTAGAGAGCGTCACTAAGTTCCACTGCGGGGTTATCTACGACTACTCCACCGCTGAGTACGTAAGCTACCGTCCGAGTGActtcggctgt                                                                                                                           >  1:452199‑M2/123‑297 (MQ=255)`  
                                                                                                                                                                                  `agagacaggaagtaaacaaggcaatatgatgggttgaaccatcaagtgactcaggggaagaagagccacactcagattcaacagactggttcaacgacacggacttctgacaggattcttgaCAGTTGTTTCAGATTAAGATATTTTTCAGTCACGATAATCAAACAGGAGAAACCATTATGATCGTGTCTGACATCGAAGCTAACGCCCTCTTAGAGAGCGGCACTAAGTTCCACTGCGGGGTTATCTACGACTACTCCACCGCTGAGTACGTAAGCTACCGTCCGAGTGACTTCAGTGc                                                                                                                            <  1:54643‑M2/179‑1 (MQ=255)`  
                                                                                                                                                                                        `cGGCTACGTCCGCTCAGTTAGTGGCAAATATGCACAAGTGTCATACATCACAGGTGTTACTCCACGCGGTGCAATCGTTGCCGATAAGACCAACATGATTCACACAGGTTTCTTGACAGTTGTTTCATATGAAGAGATTGTTAAGTCACGATAATC‑AATAGGAGAAATCAATATGATCGTTTCTGACATCGAAGCTAACGCCCTCTTAGAGAGCGTCACTAAGTTCCACTGCGGGGTTATCTACGACTACTCCACCGATGAGTACGTAAGCTACCGTCCGAGTGACTTCGGTGCGTATCTg                                                                                                                     <  1:200079/301‑1 (MQ=255)`  
                                                                                                                                                                                          `gaatacaacaaggaaaccggatggcttgaaccatcaagttactcaggggaagaagagtcacactcagagtcaacagactggtccaacgacactgacttctgacaggattcttgaCAGTTGTTTCATATGAAGAGATTGTTAAGTCACGATAATC‑AAAAGGAGAAATCAATATGATCGTTTCTGACATCGAAGCTAACGCCCTCTTAGAGAGCGTCACTAAGTTCCACTGCGGGGTTATCTACGACTACTCCACCGCTGAGTACGTAAGCTACCGTCCGAGTGACTTCGGTGCGTATCtggg                                                                                                                   >  1:370704‑M2/115‑300 (MQ=255)`  
                                                                                                                                                                                          `gaatacaacaaggaaaccggatggcttgaaccatcaagttactcaggggaagaagagtcacactcagagtcaacagactggtccaacgacactgacttctgacaggattcttgaCAGTGGTTTCATATGAAGAGATTGTTAAGTCACGATAATC‑AATAGGAGAAATCAATATGATCGTTTCTGACATCGAAGCTAACGCCCTCTTAGAGAGCGTCACTAAGTTCAACTGCGGGGTTATCTACGACTACTCCCCCGCTGATTACGTAAGCTACGGTCCGAGTGACTTCGGTGCGTATCTGGa                                                                                                                   >  1:378346‑M2/115‑301 (MQ=255)`  
                                                                                                                                                                                                                 `gcttgaaccatcaagttactcaggggaagaagagtcacactcagagtcaacagactggtccaacgacactgacttctgacaggattcttgaCAGTTGTTTCATATGAAGAGATTGTTAAGTCACGATAATC‑AATAGGAGAAATCAATATGATCGTTTCTGACATCGAAGCTAACGCCCTCTTAGAGAGCGTCACTAAGTTCCACTGCGGGGTTATCTACGACTACTCCACCGCTGAGTACGTAAGCTACCGTCCGAGTGACTTCGGTGCGTATCTGGATGCGCTGGAAGCCGAGGTTGCAc                                                                                            <  1:460944‑M2/210‑1 (MQ=255)`  
                                                                                                                                                                                                                      `aaccatcaagttactcaggggaagaagagtcacactcagagtcaacagactggtccaacgacactgacttctgacaagattcttgaCAGTTGTTTCATATGAAGAGATTGTTAAGTCACGATAATC‑AATAGGATAAATCAATCTGATCGTTTCTGACATCGAAGCTAACGCCCTCTTAGAGAGCGTCACTAAGTTCCACTGCGGGGTTATCTACGACTACTCCACCGCTGAGTACGTAAGCTACCGTCCGAGTGACTTCGGTGCGTATCTGGATGCGCTGGAAGCCGAGGTTGCACGAGGc                                                                                       <  1:349384‑M2/215‑1 (MQ=255)`  
                                                                                                                                                                                                                        `ccatcaagttactcaggggaagaagagtcacactcagagtcaacagactggtccaacgacactgacttctgacaggattcttgaCAGTTGTTTCATATGAAGAGATTGTTAAGTCACGATAATC‑AATAGGAGAAATCAATATGATCGTTTCTGACATCGAAGCTAACGCCCTCTTAGAGAGCGTCACTAAGTTCCACTGCGGGGTTATCTACGACTACTCCACCGCTGAGTACGTAAGCTACCGTCCGAGTGACTTCGGTGCGTATCTGGATGCGCTGGAAGCCGAGGTTGCACGAGGCgg                                                                                     >  1:503320‑M2/85‑301 (MQ=255)`  
                                                                                                                                                                                                                           `tcaagttactcatgggaataagagtcacactcagagtcaacagactggtccaacgacactgacttctgacaggattcttgaCAGTTGTTTCATATGAAGATATTGTTAAGTCACGATAATC‑AATAGGAGAAATAAATATGAGCGTTTCTGACATCGAAGCTAACGCCCTCTTAGAGAGCGTCACTAAGTTCCACTGCGGGGTTATCTACGACTACTCCACCGCTGAGTACGTAAGCTACCGTCCGAGTGACTTCGGGGCGTATCTGGATGCGCTGGAAGCCGAGGTTGCACGAGGCGGTCt                                                                                  <  1:221611‑M2/220‑1 (MQ=255)`  
                                                                                                                                                                                                                               `gttactcaggggaagaagagtcacacacagagtcaacagactggtccaacgacactgacttctgacaggattattgaCAGTTGTTTCATATGAAGAGATTGTTAAGTCACGATAATA‑AATAGGAGAAATAAATATGATCGTATCTGACATCGAAGCTAACGCACTATTAGAGAGAGTCACTAAGTTCCACTGCGGGGTTATCTACGAATACTCCACAGCTGAGTACGTAAGCTACAGTACGAGTGACTTAGGTGAGTATCTGGAGGCGATGGAAGCGGAGGTTGCACGGGGAGGTCTTAtt                                                                              >  1:288664‑M2/78‑301 (MQ=255)`  
                                                                                                                                                                                                                                `tttctcaggtgaagacgagtcacactcagagtcaacatactgttccaacgacactgacttctgacaggttgcttgaCAGTTGTTTCATATGAAGAGTTTGTTAAGTCACGATAATC‑AATAGGGGAAATCAATATGATTGTTTCTGACGTCGAAGCTAACGCCCTCTTAGAGAGCGTCACTAAGTTCAACTGCGGGGTTATCTACGACTACTCCACCGCTGAGTACGTAAGCTACCGTCCGAGTGACTTCGGTGCGTATCTGGATGCGCTGGAAGCCGAGGTTGCACGAGGCGGTCTTATtg                                                                             <  1:354359‑M2/225‑1 (MQ=255)`  
                                                                                                                                                                                                                                `gtgtataagagacagagagtcacactcagagtcaacagactggtccaacgacactgacttctgacaggattcttgaCAGTTGTTTCATATGAAGAGATTGTTAAGTCACGATAATC‑AATAGGAGAAATCAATATGATCGTTTCTGACATCGAAGCTAACGCCCTCTTAGAGAGCGTCACTAAGTTCCACTGCGGGGTTATCTACGACTACTCCACCGCTGAGTACGTAAGCTACCGTCCGAGTGACTTCGGTGCGTATCTGGATGCGCTGGAAGCCGAGGTTGCACGAGGCGGTCTTATtg                                                                             <  1:381499‑M2/225‑1 (MQ=255)`  
                                                                                                                                                                                                                                `cagcacaggggaagaagagtcacactcagagtcaacagactggtccaacgacactgacttctgacaggattcttgaCAGTTGTTTCATATGAAGAGATTGTTAAGTCACGATAATC‑AATAGGAGAAATCAATATGATCGTTTCTGACATCGAAGCTAACGCCCTCTTAGAGAGCGTCACTAAGTTCCACTGCGGGTTTATCTACGACTACTCCACCGCTGAGTACGTAAGCTACCGTCCGAGTGACTTCGGTGCGTATCTGGATGCGCTGGAAGCCGAGGTTGCACGAGGCGGTCTTATtg                                                                             <  1:112590‑M2/225‑1 (MQ=255)`  
                                                                                                                                                                                                                                 `tataagagacaggaagagtcacactcagagtcaacagactggtccgacgacactgacttctgacaggtttcttgaCAGTTGTTTCATATGAAGAGATTGTTAAGTCACGATAATC‑AATAGGAGAAATCAATATGATCGTTTCTGACATCGAAGCTAACGCCCTCTTAGAGAGCGTCACTAAGTTCCACTGCGGGGTTATCTACGACTACTCCACCGCTGAGTACGTAAGCTACCGTCCGAGTGACTTCGGTGCGTATCTGGATGCGCTGGAAGCCGAGGTTGCACGAGTCGGTCTTATtgt                                                                            <  1:343934‑M2/226‑1 (MQ=255)`  
                                                                                                                                                                                                                                  `actcaggggaagaagggtcacactcagagtcaacagactggtccaacgacactgacttctgacaggattcttgaCAGTTGTTTCATATGAAGAGATTGTTAAGTCACGATAATC‑AATAGGAGAAATCAATATGATCGTTTCTGACATCGAAGCTAACGCCCTCTTAGAGAGCGTCACTAAGTTCCACTGC‑GGGTTATCTACGACTACTACACCGCTGAGTACGTAAGCTACCGTCCGAGTGACTTCGGTGCGTATCTGGATGCGCTGGAAGCCGAGGTTGCACGAGGCGGTCTTATTgtgt                                                                          <  1:203956‑M2/227‑1 (MQ=255)`  
                                                                                                                                                                                                                                  `actcaggggaagaagagtcacactcagagtcaacagactggtccaacgacactgacttctgacaggattcttgaCAGTTGTTTCATATGAAGAGATTGTTAAGTCACGATAATC‑AATAGGAGAAATCAATATGATCGTTTCTGACATCGAAGCTAACGCCCTCTTAGAGAGCGTCACTAAGTTCCACTGCGGGGTTATCTACGACTACTCCACCGCTGAGTACGTAAGCTACCGTCCGAGTGACTTCGGTGCGTATCTGGATGCGCTGGAAGCCGAGGTTGCACGAGGCGTGCTTattgct                                                                           >  1:48138‑M2/75‑299 (MQ=255)`  
                                                                                                                                                                                                                                  `actcaggggaagaagagtcacactcagagtcaacagactggtccaacgacactgacttctgacaggattcttgaCAGTTGTTTCATATGAAGAGATTGTTAAGTCACGATAATC‑AATAGGAGAAATCAATATGATCGTTTCTGACATCGAAGCTAACGCCCTCTTAGAGAGCGTCACTAAGTTCCACTGCGGGGTTATCTACGACTACTCCACCGCTGAGTACGTAAGCTACCGTCCGAGTGACTTCGGTGCGTATCTGGATGCGCTGGAAGCCGAGGTTGCACGAGGCGGTCTTATtgtg                                                                           >  1:450182‑M2/75‑301 (MQ=255)`  
                                                                                                                                                                                                                                     `caggggaagaagagtcacactcggagtcaacagactggtccaacgacactgacttctgacaggattcttgaCAGTTGTTTCATATGAAGAGATTGTTAAGTCACGATAATC‑AATAGGAGAAATCAATATGATCGTTTCTGACATCGAAGCTAACGCCCTCTTAGAGAGCGTCACTAAGTTCCACTGCGGGGTTATCTACGACTACTCCACCGCTGAGTACGTAAGCTACCGTCCGAGTGACTTCGGTGCGTATCTGGATGCGCTGGAAGCCGAGGTTGCACGAGGCGGTCTTATTGTGTTc                                                                        <  1:456790‑M2/230‑1 (MQ=255)`  
                                                                                                                                                                                                                                       `gaggaagaagagtcacactcagagtcaacagactggtccaacgacactgacttctgacaggattcttgaAAGTTGTTTCATATGAAGAGATTGTTAAGTCACGAGAATA‑AATAGGAGAAATCAATATGATCGTTTCTGAGATCGAAGCTAACGCCCTCTTAGAGAGCGTCACTAAGTTCCACTGCGGGGTTATCTACGACTACTCCACCGCTGAGTACGTAAGCTACCGTCCGAGTGACTTAGGTGCGTATCTGGATGCGCTGGAAGCCGAGGTTGCACGAGGCGGTCTTATTGTGTTCca                                                                      >  1:308873‑M2/70‑301 (MQ=255)`  
                                                                                                                                                                                                                                          `gagatgtgtataagagacaggtcaacagactggtccaacgacactgacttctgacaggattcttgaCAGTTGTTTCATATGAAGAGATTGTTAAGTCACGATAATC‑AATAGGAGAAATCAATATGATCGTTTCTGACATCGAAGCAAACGCCCTCTTAGAGAGCGTCACTAAGTTCCACTGCGGGGTTATCTACGACTACTCCACCGCTGAGTACGTAAGCTACCGTCCGAGTGACTTCGGTGCGTATCTGGATGCGCTGGAAGCCGAGGTTGCACGAGGCGGTCTTATTGTGTTCCACaa                                                                   <  1:214308‑M2/235‑1 (MQ=255)`  
                                                                                                                                                                                                                                          `agagacagaCTCCACGCGGTGCAATCGTTGCCGATAAGACCAACATGTTTCACACAGGTTTCTTGACAGTTGTTTCATATGAAGAGATTGTTAAGTCACGATAATC‑AATAGGAGAAATCAATATGATCGTTTCTGACATCGAAGCTAACGCCCTCTTAGAGAGCGTCACTAAGTTCCACTGCGGGGTTATCTACGACTACTCCACCGCTGAGTACGTAAGCTACCGTCCGAGTGACTTCGGTGCGTATCTGGATGCGCTGGAAGCCGAGGTTGCACGAGGCGGTCTTATTGTGTTCCACaa                                                                   <  1:210347/293‑1 (MQ=255)`  
                                                                                                                                                                                                                                                  `gtcacactcagagtcaacagactggtccaacgacactgacttctgacagaattcttgaCAGTTGTTTCATATGAAGAGATTGTTAAGTCACGATAATC‑AATAGGAGAAATCAATATGCTCGTTTCTGACATCGAAGCTAACGCCCTCTTCGAGAGCGTCACTAAGTTCCACTGCGGGGTTATCTCCGACTACTCAACCGCTGAGTACGTAAGCTACCGTCAGAGTGACTTCGGTGCGTATCTGGATGCGCTGGACGCCGAGGGTGCACGAGGCGGTCTTATTGTGTTCCACAACGGTcaca                                                           >  1:164748‑M2/59‑301 (MQ=255)`  
                                                                                                                                                                                                                                                  `aCTCCACGCGGTGCAATCGTTGCCGATAAGACCAACATGATTCACACAGGTTTCTTGACAGTTGTTTCATATGAAGAGATTGTTAAGTCACGATAATC‑AATAGGAGAAATCAATATGATCGTTTCTGACATCGAAGCTAACGCCCTCTTAGAGAGCGTCACTAAGTTCCACTGCGGGGTTATCTACGACTACTCCACCGCTGAGTACGTAAGCTACCGTCCGAGTGACTTCGGTGCGTATCTGGATGCGCTGGAAGCCGAGGTTGCACGAGGCGGTCTTATTGTGTTCCACAACGGTcaca                                                           >  1:100278/1‑301 (MQ=255)`  
                                                                                                                                                                                                                                                      `cactcagagtcaacagactggtccaacgacactgacttctgacaggattcttgaCAGTTGTTTCATATGAAGAGATTGTTAAGTCACGATAATC‑AATAGGAGAAATCAATATGATCGTTTCTGACATCGAAGCTAACGCCCTCTTAGAGAGCGTCACTAAGTTCCACTGCGGGGTTATCTACGACTACTCCACCGCTGAGTACGTAAGCTACCGTCCGAGTGACTTCGGTGCGTATCTGGATGCGCTGGAAGCCGAGGTTGCACGAGGCGGTCTTATTGTGTTCCACAACGGTCACACGTa                                                       >  1:51218‑M2/55‑301 (MQ=255)`  
                                                                                                                                                                                                                                                        `ctcagagtcaacagactggtccaacgacactgacttctgacaggattcttgaCAGTTGTTTCATATGAAGAGATTGTTAAGTCACGATAATC‑AATAGGAGAAATCAATATGATAGTTTCTGACATCGAAGCTAACGCCCTCTTAGAGAGCGTCACTAAGTTCCACTGCGGGGTTATCAACGACTACTCCACCGCTGAGTACGTAAGCTACCGTCCGAGTGACTTCGGTGCGTATATGGATGCGCTGGAAGCCGAGGTTGCACGAGGCGGTCTTATTGTGTTCAACAACGGTCAAAAGTATg                                                     >  1:126376‑M2/53‑301 (MQ=255)`  
                                                                                                                                                                                                                                                         `aagagacagacagactggtccaacgacactgacttctgacaggattcttgaCAGTTGTATCATATGAAGAGATTGTTAAGTCACGATAATC‑AATAGGAGAAATCAATATGATCGTTTCTGACATCGAAGCTAACGCCCTCTTAGAGAGCGTCACTAAGTTCCACTGCGGGGTTATCTACGACTACTCCACCGCTGAGTACGTAAGCTACCGTCCGAGTGACTTCGGTGCGTATATGGACGCGCTGGAAGCCGAGGTTGCACGAGGCGGTCTTATTGTGTTCCACAACGGTCACAAGTATGa                                                    <  1:639‑M2/250‑1 (MQ=255)`  
                                                                                                                                                                                                                                                            `gagtcaacagactggtccaacgacactgacttctgacaggattcttgaCAGTTGTTTCATATGAAGAGATTGTTAAGTCACGATAATC‑AATAGGAGAAATCAATATGATCGTTTCTGACATCGAATCTAACGCCCTCTTAGAGAGCGTCACTAAGTTCCACTGCGGGGTTATCTACGACTACTCCACCGCTGAGTACGTAAGCTACCGTCCGAGTGACTTCGGTGCGTATCTGGATGCGCTGGAAGCCGAGGTTGCACGAGGCGGTCTTATTGTGTTCCACAACGGTCACAAGTATGACGt                                                 >  1:139320‑M2/49‑301 (MQ=255)`  
                                                                                                                                                                                                                                                                `caacagactggtccaacgacactgacttctgacaggattcttgaCAGTTGTTTCATATGAAGAGATTGTTAAGTCACGATAATC‑AATAGGAGAAATCAATATGATCGTTTCTGACATCGAAGCTAACGCCCTCTTAGAGAGCGTCACTAAGTTCCACTGCGGGGTTATCTACGACTACTCCACCGCTGAGTACGTAAGCTACCGTCCGAGTGACTTCGGTGCGTATCTGGATGCGCTGGAAGCCGAGGTTGCACGAGGCGGTCTTATTGTGTTCCACAACGGTCACAAGTATGACGTTCCt                                             >  1:263653‑M2/45‑301 (MQ=255)`  
                                                                                                                                                                                                                                                                     `gactggtccaacgacactgacttcttacaggattcttgaCAGTTGTGTCATATGAAGAGATTGTTAAGTCACGATAATCAAACAGGAGAAATCAATATGATCGTTTCTGACATCGAAGCTAACGCCCTCTTAGAGAGCGTCACTAAGTTCCACTGCGGGGTTATCTACGACTACTCCACCGCTGAGTACGTAAGCTACCGTCCGAGGGACTTCGGTGCGTATCTGGATGCGCTGGAAGCCGAGGTTGCACGAGGCGGTCTTATTGTGTTCCACAACGGTCACAAGTATGACGTTCCTGCAt                                         <  1:314739‑M2/262‑1 (MQ=255)`  
                                                                                                                                                                                                                                                                         `ggtccaacgacactgacttctgacaggattcttgaCAGTTGTTTCATATGAAGAGATTGTTAAGTCACGATAATC‑AATAGGAGAAATCAATATGATCGTTTATGACATCGAAGCTAACGCCCTCTTAGAGAGCGTCACTAAGTTCCACTGCGGGGTTATCTACGACTACTCCACCGCTGAGTACGTAAGCTACCGTCCGAGTGACTTCGGTGCGTATCTGGATGCGCTGGAAGCCGAGGTTGCACGAGGCGGTCTTCTTGTGTTCCACAACGGTCACAAGTATGAAGTTCCTGCATTGAcc                                    >  1:115733‑M2/36‑301 (MQ=255)`  
                                                                                                                                                                                                                                                                             `aaGACAAACATGAATCACACAGGTTTCTTGACAGTTGTTTCATATGAAGAAATTGTTAAGTCACGATAATT‑AATAGGAGAAATCAAGATGATCGTTTCTGACATCGAAGCTAACGCCCTCTTAGAGAGCGTCACTAAGTTCCACTGCTGGGTTATCTACGACTACTCCACCGCTGAGTACGTAAGCTACCGTCCGAGTGACTTCGGTGCGTATCTGGATGCGCTGGAAGCCGAGGTTGCACGAGGCGGTCTTATTGTGTTCCACAACGGTCACAAGTATGACGTTCCTGCATTGACCAAAc                                <  1:448807/301‑1 (MQ=255)`  
                                                                                                                                                                                                                                                                              `aGACCAACATGATTCACACAGGTTTCTTGACAGTTGTTTCATATGAAGAGATTGTTAAGTCACGATAATC‑AATAGGAGAAATCAATATGATCGTTTCTGACATCGAAGCTAACGCCCTCTTAGAGAGCGTCACTAAGTTCCACTGCGGGGTTATCTACGACTACTCCACCGCTGAGTACGTAAGCTACCGTCCGAGTGACTTCGGTGCGTATCTGGATGCGCTGGAAGCCGAGGTTGCACGAGGCGGTCTTATTGTGTTCCACAACGGTCACAAGTATGACGTTCCTGCAtctgtctcttt                               >  1:366824/1‑291 (MQ=255)`  
                                                                                                                                                                                                                                                                                `agacactgacttctgacaggattcttgaCAGTTGTTTCATATTAAGAGATTGTTAAGTCACGATAATC‑AATAGGAGAAATCAATATGATCGTTTCTGACATCGAAGCTAACGCCCTCTTAGAGAGCGTCACTAAGTTCCACTGCGGGGTTATCTACGACTACTCCACCGCTGAGTACGTAAGCTACCGTCCGAGTGACTTCGGTGCGTATCTGGATGCGCTGGAAGCCGAGGTTGCACGAGGCGGTCTTATTGTGTTCCACAACGGTCACAAGTATGACGTTCCTGCATTGACCAAACTgg                             >  1:505563‑M2/29‑301 (MQ=255)`  
                                                                                                                                                                                                                                                                                  `acactgacttttgacaggattcttgaCAGTTGTTTCATATGAAGAGATTGTTAAGTCACGATAATC‑AATAGGAGCAATCAATATGATCGTTTCTGACATCGAAGCTAACGCCCTCTTAGAGAGCGTCACTAAGTTCCACTGCGGGGTTATCTACGAATACTCCACCGCTGAGTACGTAAGCTACCGTCCGAGTGACTTCGGTGCGTAACTGGATGCTCTGGAAGCCGAGGTTGCACGAGGCGGTCTTATTGTGTTCCACAACGGTCACAAGTATGCCGTTCCTGCCTTGACCAAACTGGCa                           >  1:269187‑M2/27‑301 (MQ=255)`  
                                                                                                                                                                                                                                                                                   `cactgacttctgacaggattcttgaCAGTTGTTTCATATGAAGAGATTGTTAAGTCACGATAATC‑AATAGGAGAAATCAATATGATCGTTTCTGACATCGAAGCTAACGCCCTCTTAGAGAGCGTCACTAAGTTCCACTGCGGGGTTATCTACGACTACTCCACCGCTGAGTACGTAAGCTACCGTCCGAGTGACTTCGGTGCGTATCTGGATGCGCTGGAAGCCGAGGTTGCACGAGGCGGTCTTATTGTGTTCCACAACGGTCACAAGTATGACGTTCCTGCATTGACCAAACTGGCaa                          >  1:258804‑M2/26‑301 (MQ=255)`  
                                                                                                                                                                                                                                                                                      `gtccaacgacactgacttctgaCAGTTGTTTCATATGAAGAGATTGTTAAGTCACGATAATC‑AATAGGAGAAATCAATATTATCGTTTCTGACATCGAAGCTAACGCCCTCTTAGAGAGCGTCACTAAGTTCCACTGCGGGGTTATCTACGACTACTCCACCGCTGAGTACGTAAGCTACCGTCCGAGTGACTTCGGTGCGTATCTGGATGCGCTGGAAGCCGAGGTTGCACGAGGCGGTCTTATTGTGTTCCACAACGGTCACAAGTATGACGTTCCTGCATTGACCAAACTGGCAAAGt                       >  1:91382‑M2/23‑301 (MQ=255)`  
                                                                                                                                                                                                                                                                                        `acttctgacaggattctttaCAGTTGTTTCTTATGAAGAGATTGTTAAGTCACGATAATC‑AATAGGAGAAATCAATATCATCGTTTCTGACATCGAAGCTAACGCCCTCTTAGAGAGCGTCACTAAGTTCCACTGCGGGGTTATCTACGACTTCTCCACCGCTGAGTACGTAAGCTACCGTCCGAGTGACTTCGGTGCGTATCTGGATGCGCTGGAAGCCGAGGTTGCACGAGGCGGTCTTATTGTGTTCCACAACGGTCACAAGTATGACGTTCCTGCATTGACCAAACTGGCAAAGTTg                     <  1:28277‑M2/281‑1 (MQ=255)`  
                                                                                                                                                                                                                                                                                         `cttctgacaggattcttgaCAGTTGTTTCATATGAAGAGATTGTTAAGTCACGATAATC‑AATAGGAGAAATCAATATGATCGTTTCTGACATCGAAGCTAACGCCCTCTTAGAGAGCGTCACTAAGTTCCACTGCGGGGTTATCTACGACTACTCCACCGCTGAGTACGTAAGCTACCGTCCGAGTGACTTCGTTGCGTATCTGGATGCGCTGGAAGCCGAGGTTGCACGAGGCGGTCTTATTGTGTTCCACAACGGTCACAAGTATGACGTTCCTGCATTGACCAAACTGGCAAAGTTGc                    >  1:358686‑M2/20‑301 (MQ=255)`  
                                                                                                                                                                                                                                                                                              `gacaggattcttgaCAGTTGTTTCATATGAAGAGATTGTTAAGTCACGATAATC‑AATAGGAGAAATCAATATGATCGTTTCTGACATCGAAGCTAACGCCCTCTTAGAGAGCGTCACTAAGTTCCACTGCGGGGTTATCTACGACTACTCTACCGCTGAGTACGTAAGCTACAGTCCGAGTGACTTCGGTGCGTATCTGGATGCGCTGGAAGCCGAGGTTGCACGCGGCGGTCTTATTGTGTTCCAAAACGGTCACAAGTATGACGTTCCTGCATTTACCAAACTGGCAAAGTTGCAATTg               >  1:467139‑M2/15‑301 (MQ=255)`  
                                                                                                                                                                                                                                                                                                   `gattcttgaCAGTTGTTTCATATGAAGAGATTGTTAAGTCACGATAATC‑AATAGGAGAAATCAATATGATCGTTTCTGACATCGAAGCTAACGCCCTCTTAGAGAGCGTCACTAAGTTCCACTGCGGGGTTATCTACGACTACTCCACCGCTGCGTACGTAAGCTACCGTCCGAGTGACTTCGGTGCGTATCTGGATGCGCTGGAAGCCGAGGTTGCACGAGGCGGTCTTATTGTGTTCCACAACGGTCACAAGTATGACGTTCCTGCATTGACCTAACTGGCAAAGTTGCAATTGAACCg          >  1:428958‑M2/10‑301 (MQ=255)`  
                                                                                                                                                                                                                                                                                                   `gattcttgaCAGTTGTTTCATATGAAGAGATTGTTAAGTCACGATAATC‑AATAGGAGAAATCAATATGATCGTTTCTGACATCGAAGCTAACGCCCTCTTAGAGAGCGTCACTAAGTTCCACTGCGGGGTTATCTACGACTACTCCACCGCTGAGTACGTAAGCTACCGTCCGAGTGACTTCGGTGCGTATCTGGATGCGCTGGAAGCCGAGGTTGCACGAGGCGGTCTTATTGTGTTCCACAACGGTCACAAGTATGACGTTCCTGCATTGACCAAACTGGCAAAGTTGCAATTGAACCg          >  1:275808‑M2/10‑301 (MQ=255)`  
                                                                                                                                                                                                                                                                                                       `cttgaCAGTTGTTTCATATGAAGAGATTGTTAAGTCACGATAATC‑AATAGGAGAAATCAATATGATCGTTTCTGACATCGAAGCTAACGCCCTCTTAGAGAGCGTCACTAAGTTCCACTGCGGGGTTATCTACGACTACTCCACCGCTGCGTACGTAAGCTACCGTCCGAGTGACTTCGGTGCGTATCTGGATGTGCTGGAAGCCGAGGTTGCACGAGGCGGTCTTATTTTGTTCCACAACGGTCACAAGTATGACGTTCCTGCATTGACCAAACTGGCAAAGTTGCAATTGAACCGagag      >  1:387918‑M2/6‑301 (MQ=255)`  
                                                                                                                                                                                                                                                                                                        `ttgaCAGTTGTTTCATATGAAGAGATTGTTAAGTCACGATAATC‑AATAGGAGAAATCAATATGATCGTTTCTGACATCGAAGCTAACGCCCTCTTAGAGAGCGTCACTAAGTTCCACTGCGGGGTTATCTACGACTACTCCACCGCTGAGTACGTAAGCTACCGTCCGAGTGACTTCGGTGCGTATCTGGATGCGCTGGAAGCCGAGGTTGCACGAGGCGGTCTTATTGTGTTCCACAACGGTCACAAGTATGACGTTCCTGCATTGACCAAACTGGCAAAGTTGCAATTGAACCGAGAGt     <  1:441536‑M2/297‑1 (MQ=255)`  

                                                                                                                                                                                                                                                                                                            `|`                                                                                                                                                                                                                                                                                                              
`ATGAAGCTAAGACTCGCTCCGCTGTTCACATCTTGAAGAACTTAGGCTGGACTTGGACAAGACAGACTGGCTGGAAGAAACCAGAAGTTACCAAGCTGAGTCATAAGGTGTTCGATAAGGACACTATGACCCACATCAAGGCTGGTGATTGGGTTAAGGTTGACATGGGAGTTGTTGGTGGATACGGCTACGTCCGCTCAGTTAGTGGCAAATATGCACAAGTGTCATACATCACAGGTGTTACTCCACGCGGTGCAATCGTTGCCGATAAGACCAACATGATTCACACAGGTTTCTTGACAGTTGTTTCATATGAAGAGATTGTTAAGTCACGATAATC‑AATAGGAGAAATCAATATGATCGTTTCTGACATCGAAGCTAACGCCCTCTTAGAGAGCGTCACTAAGTTCCACTGCGGGGTTATCTACGACTACTCCACCGCTGAGTACGTAAGCTACCGTCCGAGTGACTTCGGTGCGTATCTGGATGCGCTGGAAGCCGAGGTTGCACGAGGCGGTCTTATTGTGTTCCACAACGGTCACAAGTATGACGTTCCTGCATTGACCAAACTGGCAAAGTTGCAATTGAACCGAGAGTTCC  >  NC_001604.1/13997‑14596`

  

Alignment Legend

Aligned base mismatch/match (shaded by quality score): `ATCG/ATCG` `< 3 ≤` `ATCG/ATCG` `< 10 ≤` `ATCG/ATCG` `< 21 ≤` `ATCG/ATCG` `< 34 ≤` `ATCG/ATCG` `< 38 ≤` `ATCG/ATCG`

Unaligned base: `atcg`    Masked matching base: `atcg`    Alignment gap: `‑`    Deleted base: `‑`

Reads not counted as support for junction

read\_name Not counted due to insufficient overlap past the breakpoint.

read\_name Not counted due to not crossing MOB target site duplication.
